# Salicine in Colliquative Diarrhea

**Published:** 1873-10

**Authors:** J. D. Tucker

**Affiliations:** Jackson, Tenn.


### SALICINE IN COLLIQUATIVE DIARRHEA.

BY J. D. TUCKER, M. D., JACKSON, TENN.

July 5th, I was called to Mr. B. N., aged 65, who was attacked at 10 o'clock the night before with a violent pain and cramping in the stomach, bowels and lower extremities. Friction and hot application relieved him for two or three hours, when a very copious and watery discharge from the bowels set in, which soon exhausted the patient.

I reached him at 9 A. M.; found him free from pain and cramp, abdomen shrunken but not tender, pulse low, tongue natural. I ordered brandy every three or four hours, with a pill composed of ½ gr. each of opium, ipecac, camphor, blue mass and quinine, every two hours until the bowels were checked; chicken tea and rice, if he should desire any food, which he did not.

On the morning of the 6th he sent me a report that he was doing well.

7th. Called at 4 o'clock, A. M.; found him prostrated, bowels moving continuously; pulse 80; tongue cold, but looked natural; extremities cold; irritable stomach, though no pain nor tenderness. Ordered brandy freely, and gave a solution of morphine, creosote and mint, every hour, with sinapism to epigastrium, until the stomach was quieted. Then ordered opium with act. lead every 2 hours for 12 hours, but did no good. I then tried tannin, with anodynes, *heavy;* alternating with small doses of mercury and chalk; all to no use. I finally tried all the important astringents without the least benefit.

The 9th found the patient near the grave, and me without a hope of success. During these two days the patient had taken nothing as food or drink, except a little rice water and powdered ice, as there was extreme thirst; but everything taken would pass away in less time than an hour. Tongue now pale and cold; pulse yet 80. No tenderness; features cadaveric; extremities shrivelled, and stomach rejecting everything.

At this point in the case, I rode home for recreation, thinking I might, while absent from the excitement of relations and friends, study up something that would stay the trouble. When I reached my office, I took up "Naphey's Modern Therapentisy," and looked over his treatment for diarrhea, and to my great satisfaction, I found a quotation from Prof. Aitkin's Practice, which I felt sure would meet the indications in my case. I immediately fixed up the prescription and returned to the patient, and to my great astonishment, found him still alive. I gave a dose right away, and sat down by the sick man to watch its effects. In the space of one hour his tongue was getting warm, face perspiring, skin feeling natural, eyes looking brighter, stomach quieting, bowels slightly checking up, &c. In two hours more all the symptoms were still better. I gave another dose, and ordered a repetition of the same every five hours, with ice and mutton tea; but he took neither tea nor ice for twenty-four hours. By the second day from the beginning of this treatment, all the bad symptoms had entirely subsided, and the appetite was returning. On the third day I dismissed him doing well. He improved steadily from the first dose, which was five grains of salicine, repeated every five hours for three days, with no other medicine.

What Prof. Aitkin says may not be new to the profession, but it was to me.

